# Salivary Proteins Associated with Periodontitis in Patients with Type 2 Diabetes Mellitus

**DOI:** 10.3390/ijms13044642

**Published:** 2012-04-12

**Authors:** Hang Haw Chan, Zubaidah H. A. Rahim, Kala Jessie, Onn H. Hashim, Tara B. Taiyeb-Ali

**Affiliations:** 1 Department of Oral Biology, Faculty of Dentistry, University of Malaya, 50603 Kuala Lumpur, Malaysia; E-Mails: chanhh0110@gmail.com (H.H.C.); jessiekala7@yahoo.com (K.J.); 2 Department of Molecular Medicine, Faculty of Medicine, University of Malaya, 50603 Kuala Lumpur, Malaysia; E-Mail: onnhashim@um.edu.my; 3 University of Malaya Centre for Proteomics Research, Faculty of Medicine, University of Malaya, 50603 Kuala Lumpur, Malaysia; 4 Department of Oral Pathology and Oral Medicine and Periodontology, Faculty of Dentistry, University of Malaya, 50603 Kuala Lumpur, Malaysia; E-Mail: tara@um.edu.my

**Keywords:** periodontitis, type 2 diabetes mellitus, salivary protein, 2-DE

## Abstract

The objective of this study was to investigate the salivary proteins that are associated with periodontitis in patients with Type 2 diabetes mellitus (T2DM). Volunteers for the study were patients from the Diabetic Unit, University of Malaya Medical Centre, whose periodontal status was determined. The diabetic volunteers were divided into two groups, *i.e.*, patients with periodontitis and those who were periodontally healthy. Saliva samples were collected and treated with 10% TCA/acetone/20 mM DTT to precipitate the proteins, which were then separated using two-dimensional polyacrylamide gel electrophoresis. Gel images were scanned using the GS-800^TM^ Calibrated Densitometer. The protein spots were analyzed and expressed in percentage volumes. The percentage volume of each protein spot was subjected to Mann-Whitney statistical analysis using SPSS software and false discovery rate correction. When the expression of the salivary proteins was compared between the T2DM patients with periodontitis with those who were periodontally healthy, seven proteins, including polymeric immunoglobulin receptor, plastin-2, actin related protein 3, leukocyte elastase inhibitor, carbonic anhydrases 6, immunoglobulin J and interleukin-1 receptor antagonist, were found to be differentially expressed (*p* < 0.01304). This implies that the proteins may have the potential to be used as biomarkers for the prediction of T2DM patients who may be prone to periodontitis.

## 1. Introduction

Diabetes mellitus is a major global health problem and it has an increasing prevalence due to several factors, such as population growth, aging, urbanization and increasing prevalence of obesity or lack of physical exercise. The number of people diagnosed with diabetes is increasing at an alarming rate. It is estimated that by the year 2030, 366 million people worldwide will have the disease [1]. Based on the Diabetes Atlas [2], the highest numbers of patients are currently found mainly in western countries, such as in the European region and the Western Pacific region. This, however, will change in the year 2025 where the greatest number of diabetic patients is expected to be from the Asian region [2,3]. In Malaysia, Ministry of Health studies estimate that the number of people having diabetes was more than 10% of the total population (26.64 millions) in 2006 [4], and that this number is estimated to increase every year. In the US alone, at least 8.3% of the total population has diabetes mellitus with 18.8 million people diagnosed, and an estimated further 7 million undiagnosed. Diabetes mellitus is the seventh leading cause of death in the US. In Malaysia, it was estimated that for the year 2000, at least 2261 deaths were caused by diabetes mellitus [5].

Diabetes mellitus is the leading cause of heart disease, stroke, hypertension, blindness and other eye problems, kidney disease, amputation and oral diseases. Periodontitis has been considered as the sixth complication of type 2 diabetes mellitus (T2DM) after retinopathy, nephropathy, neuropathy, cardiovascular disease and peripheral vascular disease [6]. It had been reported in several studies that patients with T2DM have an increased risk of periodontitis [7–9]. Periodontitis is an inflammatory disease of the periodontium, primarily caused by bacterial infection [10]. In advanced cases of periodontitis, degradation of the connective tissue and destruction of the bone occur, ultimately leading to tooth loss in adults [11]. Current evidence shows that diabetes and persisting hyperglycemia will lead to exaggerated immune-inflammatory response to the periodontal bacterial challenges, which will result in more rapid and severe periodontal tissue destruction [10].

Poor metabolic control as well as an extended period of diabetic status are risk factors for periodontitis when abundant dental biofilm is present on the teeth [8]. Grossi *et al.*, [12] have reported that effective management of periodontal infection by utilizing systemic antibiotics can be beneficial to diabetic patients. These antibiotics will reduce the local signs and symptoms of periodontitis as well as improve the diabetic status of the patient. They recommend that control of periodontal disease can lead to improvement of the diabetic status of the patient. In the National Oral Health Survey of adults undertaken by the Oral Health Division, Ministry of Health, Malaysia [13], there was a high prevalence of periodontal disease in the adult population. About 90% of the population aged between 45 and 70 apparently had periodontal problems.

To date, there is no data from any studies regarding patients with diabetes and periodontitis using the proteomic approach. In this study, the profiles of proteins in saliva samples from volunteers suffering from T2DM with periodontitis were compared with those of diabetics with a healthy periodontium. Saliva, and not blood, was chosen as the sample used in the study, as many reports have suggested that saliva can be an alternative to blood [2]. Saliva contains a large number of proteins which have metabolic, immune response, transporting and several other cellular functions [14–18]. Its collection is non-invasive compared to the collection of other body fluids, and hence has a great potential for use in the diagnosis of systemic and localized diseases. The proteins in the saliva samples were separated using two-dimensional polyacrylamide gel electrophoresis (2-DE) method and analyzed for any differential expression which may have the potential as predicting factors for the development of periodontitis in T2DM individuals.

## 2. Results and Discussion

Human whole saliva contains fluids, mainly from the salivary glands as well as contributions from the gingival crevicular fluid, oral tissues, epithelial cells, bronchial and nasal secretions, and traces of blood, bacteria and viruses. Compared to serum, whole saliva is consistently under the influence of a hostile environment, with its proteins subjected to changes and modifications by host- and foreign-derived enzymes [19]. This results in the possible generation and modifications of proteins in whole saliva.

**Figure 1:**
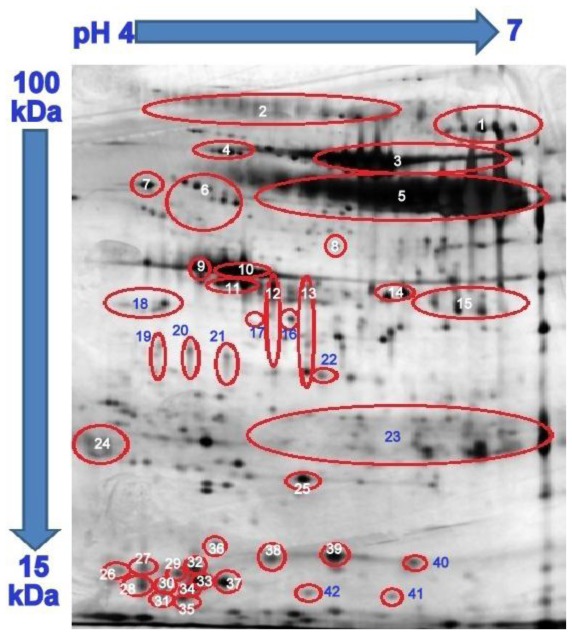
Typical 2-DE profile of human saliva protein. Comparisons were made with the one published by Jessie *et al.* [23] and the protein spots were suspected to be those as listed in Table 1.

In the present study, dry strips of 11 cm length and pH range of 4 to 7 were used. This range of pH was chosen as most of the saliva proteins were resolved in acidic pH between 4 to 7 [20]. The 2-DE profiles of whole saliva proteins from T2DM patient volunteers with periodontitis and T2DM patients with healthy periodontium that were generated in this study (Figure 1) showed a strong resemblance to those which were previously reported [20–24]. This is most likely due to similarities in the subjects that were involved in the studies (race, age range and inclusion and exclusion criteria) as well as the methodology that was used. By comparing the 2-DE profiles generated in this study with those reported by Jessie *et al.* [23], most of the proteins could be identified. There were 42 highly resolved spots that could be matched to a total of 23 different proteins (Table 1).

Analysis by densitometry detected the differential expression of seven salivary proteins between the two groups of T2DM patients. This include protein immunoglobulin J (IGJ) chain (+1.743; *p* < 0.001), polymeric immunoglobulin receptor (pIgR) (−1.344; *p* = 0.008), plastin-2 (PLS2) (+2.381; *p* < 0.001), actin-related protein (Arp) (−5.802; *p* = 0.001), interleukin-1 receptor antagonist (IL-1ra) (−4.132; *p* < 0.001), leukocyte elastase inhibitor (LEI) (+1.919; *p* = 0.004) and carbonic anhydrase VI (CA VI) (−1.365; *p* = 0.012) (Table 2).

IGJ exhibited an increase of 1.7-fold expression in the T2DM patients with periodontitis compared to the controls. The IGJ chain is an 18 kDa protein. It is produced and excreted by mucosal and glandular plasma cells which regulate the polymer formation of IgA [25] and IgM [26–28]. IGJ helps these immunoglobulins to bind to the secretory component. It provides the polymeric IgA and pentameric IgM with the capacity to bind pIgR and this subsequently regulates the pIg structure [26,28]. The pIgR mediates the active transport of bound pIg from the basolateral to the apical face of exocrine epithelial cells, thus releasing secretory antibodies to the mucosal surfaces [26,28]. This may provide an explanation for the high levels of IGJ in the saliva from the T2DM patients with periodontitis. Secretory IgA (sIgA) and secretory IgM antibodies, which serve as the first line of specific immunologic defense, are actively transported to the mucosal secretions via the IGJ.

Contrariwise, pIgR appears to be down-regulated in T2DM patients with periodontitis. This is not quite understood. Nevertheless, studies have shown that proteins may be recycled, degraded or transcytosed to the opposite surface after delivery to one surface [29].

In this study, the expression of plastin-2 (PLS2) was found to be higher in the T2DM patients with periodontitis compared to the group with healthy periodontium. This observation is in agreement with the report of Bostanci *et al.* [30], which suggests that PLS2 is associated with periodontitis. PLS2, which is also known as L-plastin (LCP1), has a role in the regulation of leukocyte adhesion [31], suggesting that many signaling events implicated in integrin regulation activities via induction of L-plastin phosphorylation [32]. PLS2 belongs to the actin-binding protein family, which is found in cells of hematopoetic origin, such as leukocytes. It has been reported that the high concentration of this protein in gingival crevicular fluid (GCF) facilitates recruitment of polymorphonuclear neutrophils (PMN) at sites of inflammation. This is in agreement with the report on the potential constitutive PMN hyper-reactivity in non-diabetic volunteers with periodontitis [30]. In another study, it was shown that there was a reduction in PLS2 in the GCF of non-diabetic volunteers with gingivitis, suggesting that there is a less adherent phenotype in neutrophils during the stage of inflammatory response [33].

Leukocyte elastase inhibitor (LEI) is a 43 kDa protein that was also shown to be up-regulated in patients with T2DM who had periodontitis in this study. LEI is a naturally occurring inhibitor of neutrophil proteases [34]. The imbalance of proteases and their natural inhibitors due to the excess release by neutrophils and monocytes is thought to be responsible for tissue injury in human inflammatory diseases such as respiratory disease, joint inflammation, sepsis and skin diseases [35]. LEI has been reported to function as a physiological inhibitor of the proteases that are important in the immune defense but when present in excess, they function as major agents of inflammation by destroying matrix proteins as well as immune defense molecules. The higher amount of LEI in diabetics with periodontitis observed in this study suggests that this protein may be responsible for further destruction of the periodontium, matrix proteins, further lowering the immune defense system and subsequently, destruction of the alveolar bone.

The CO_2_-carbonic acid-bicarbonate system is responsible for most of the buffering capacity in the human whole saliva. The salivary glands are able to produce bicarbonate from CO_2_, yielding salivary bicarbonate levels that are usually slightly lower than plasma levels [36]. Carbonic anhydrases (CAs) catalyze the reversible reaction of CO_2_ + H_2_O ↔ HCO_3_ − + H^+^. There are several carbonic anhydrase isoenzymes, with CA II and CA VI being mainly expressed in human salivary glands, where CA VI is secreted in the saliva [37–39]. A recent study had shown that the low level of salivary CA VI expression is associated with an increased risk of caries [39]. It has been reported that the saliva CA VI accumulates in the enamel pellicle maintaining its enzymatic activity, and that it may catalyze the neutralization of the acid produced by bacteria, thus providing an immune defense locally on tooth surfaces [39]. The lower level of CA VI that was observed in the T2DM patients with periodontitis suggests its higher risk in the development of caries and possibly periodontitis.

Actin-related protein 3 (Arp3) functions as an ATP binding component of the Arp2/3 complex [40]. In the present study, Arp 3 appeared to have lower expression in T2DM patients with periodontitis compared to the controls. Arp2/3 complex builds a network of actins to help the movement of immune cells and enables them to guard against bacterial infection [41,42]. When production of Arp3 is lowered, the binding of ATP to produce the Arp2/3 complex is also reduced, making the host more susceptible to infection.

The higher expression of interleukin-1 receptor antagonist (IL-1Ra) in the T2DM group with healthy periodontium suggests its possible role in protecting the periodontium from the inflammatory effect of IL-1. IL-1 is a type of proinflammatory protein that inhibits the function β cells and induces apoptosis. The body secretes IL-1Ra to counter the effect of IL-1. It is a naturally occurring anti-inflammatory protein capable of inhibiting both types of IL-1 signaling and protecting the β cells [43–49]. In patients with T2DM it is a common situation that the functional β cell mass is unable to compensate for the increasing insulin need due to insulin resistance. In the course of the disease, the β cells lose its ability to function, irrespective of the treatment given. In T2DM patients with periodontitis, inflammation may have already occurred in the periodontium, leading to a lower level of IL-1Ra expression to counter the inflammation.

## 3. Experimental Section

### 3.1. Collection and Pre-Treatment of the Saliva Samples

The current study was carried out with the approval of the Ethical Committee (Institutional Review Board) of the Faculty of Dentistry, University of Malaya (Ethics approval DFOB1003/0034(P)). Patients who had been confirmed with T2DM (WHO 1999) from the Diabetic Clinic of the University of Malaya Medical Centre and were on regular follow-up for a minimum of 6 months and who gave their written consent were enrolled into the study. The subjects, aged between 40 and 60 years, were screened by BPE/CPI using the WHO probe. Periodontally-healthy T2DM patients (Code 0) and those with periodontitis (Codes 3 & 4) were selected. The periodontitis subjects were reassessed and those with a minimum of 12 teeth were selected. Patients finally recruited for the study were those with ≤5 pockets of ≤5 mm probing depth and probing attachment loss of ≤4 mm in at least two different quadrants which bled on probing. Periodontal assessments were performed using the Florida Probe. Patients were excluded if they fit into any of the following categories: a history of systemic antibiotic usage over the previous 4 months; periodontal treatment within the past 6 months; were current smokers; pregnant, and had a cerebrovascular or cardiovascular event within the past 12 months. A single periodontal examiner, a resident in the Department of Periodontology had Kappa agreement values >0.81 in his intra-examiner reproducibility study: for plaque (0.88), gingival bleeding (0.86), probing pocket depth (0.96) and probing attachment loss (0.96), prior to the study.

The saliva of 10 periodontally healthy T2DM subjects and 15 selected T2DM patients with periodontitis was collected. Volunteers were advised to refrain from eating, drinking, smoking and performing any oral hygiene activity for at least 1 hour before saliva collection. During the saliva collection, volunteers were advised not to talk or move their tongue in the mouth. Saliva collection was usually done in the morning between 9 a.m. and 11 a.m. [24] by spitting into an ice-chilled tube which contained a complete protease inhibitor cocktail at a final concentration recommended by the manufacturer (Roche Ltd, Basel, Switzerland) [20]. Samples were mixed thoroughly before being kept in an ice box and transported to the laboratory for processing. The saliva samples were then centrifuged at 14,000 g for 20 minutes at 4 °C in order to remove any debris, cells and food particles present. Aliquots of 500 μL saliva samples were then transferred to smaller tubes and 500 μL of 10% of TCA and 10 μL 20 mM DTT in acetone was added to precipitate the protein [20]. Precipitated protein samples were stored at −80 °C until used in the 2-DE analysis. Analysis by 2-DE was performed on the 25 saliva samples individually in triplicates giving rise to 75 2-DE gels to be analyzed.

### 3.2. First Dimensional Separation—Isoelectric Focusing

Precipitated protein (140 ug) was dissolved in 500 μL rehydration buffer containing 7 M urea, 2 M thiourea, 65 mM DTT, 4% CHAPS, 0.5% IPG Buffer and 0.002% bromophenol blue and quantified using the Bradford protein assay kit (Bio-Rad, Hercules, CA, USA) according to the manufacturer’s instructions. The protein solution was then applied onto 11 cm rehydrated precast immobiline dry strips with pH 4–7 (GE Healthcare BioSciences, Uppsala, Sweden). The dry strips were allowed to rehydrate passively for 12 hours before being subjected to first dimensional separation by isoelectric focusing (IEF) using the Bio-Rad Protean IEF system (Bio-Rad, Hercules, CA, USA).

### 3.3. Second Dimensional Separation—Polyacrylamide Gel Electrophoresis

Polyacrylamide gels were prepared as previously described [20]. Second dimensional polyacrylamide gel electrophoresis was performed on Bio-Rad Protean II XL Cell (Bio-Rad, Hercules, CA, USA) gel tank using the manufacturer’s recommended conditions.

All of the 75 2-DE gels were then developed and visualized by silver staining [50].

### 3.4. Scanning and Analysis of Gel Image

A GS-800^TM^ Calibrated Densitometer (Bio-Rad, Hercules, CA, USA) was used to scan and store images of the 2-DE gels. Quantity One and PDQuest computer softwares (Bio-Rad, Hercules, CA, USA) were used to evaluate protein profiles and perform protein analyses. The percentage volume contribution (% vol) of a protein was used to determine proteins that were differentially expressed in the saliva. The percentage volume contribution of a protein spot refers to the spot volume of a protein expressed as a percentage of the total spot volume of all the salivary proteins that was detected in the gel. Data obtained from this method was free from variations attributed to protein loading and staining intensity. The protein spots were expressed as mean ± standard error of means (SEM) and their rate of occurrence was also determined.

### 3.5. Identification of Saliva Proteins

Several protein spots of interest were excised from the polyacrylamide gels and identified using electrospray (LC/MS/MS) mass spectrometry with automatic database analysis (Proteomics International, Perth, Australia). After confirming the protein identity, other 2-DE protein profiles obtained were compared visually with those published [20–24].

### 3.6. Statistical Analysis

Statistical analysis was done using the IBM SPSS statistics, version 19 (IBM Corporation: Chicago, IL, USA, 2010). Comparison of the protein profiles between T2DM patients with periodontitis and T2DM patients with no periodontitis relates protein changes that are associated with periodontitis. Expression of saliva proteins of the two groups of T2DM patients were analyzed using the Mann-Whitney statistical analysis for significant differences. False discovery rate correction for the *p* value was done using a previously described method [51]. Fold changes calculation was done according to the Equation (1):

(1) $$\frac{\text{Pr}\;oteins\;in\;Diabetics\;with\;periodontitis}{Similar\;protein\;in\;Diabetics\;with\;healthy\;periodontium\;}=Fold\;changes(\geq +1.00)$$

For the fold changes which are less than 1, the Equation (2) was used:

(2) $$\frac{1}{Fold\;changes\;(≺+1.00)}=Fold\;changes\;(-1.00)$$

## 4. Conclusions

Comparative 2-DE profiling of the saliva samples of T2DM patients with periodontitis and those without periodontitis demonstrated the significant differential expression of seven proteins. Whilst the expression of pIgR, Arp 3, CA VI and IL-1Ra was down-regulated, PLS-2, LEI and IGJ chain appeared to be up-regulated in the saliva of the T2DM patients with periodontitis compared to the controls. Aside from being involved in the physiological response towards periodontitis, these proteins have the potential to be used as biomarkers for the prediction of T2DM patients who may be prone to periodontitis.

## Figures and Tables

**Table 1:** Densitometry analysis of saliva proteins and their rates of presence in protein profiles.

Proteins	Protein Label	Diabetes with Periodontitis % Volume a (±SEM)	RP b/15	Diabetes with Healthy Periodontium % Volume a (±SEM)	RP b//10
Serotransferrin	1	2.602 (±1.274)	15	3.430 (±1.254)	10
pIgR	2	5.093 (±1.591)	15	6.848 (±1.273)	10
Plastin 2	4	0.434 (±0.161)	15	0.182 (±0.039)	8
Alpha-1-antitrypsin	6	1.858 (±0.641)	15	1.818 (±0.836)	10
Protein disulfide isomerase	7	0.230 (±0.090)	14	0.199 (±0.093)	8
Actin-related protein 3	8	0.023 (±0.004)	14	0.135 (±0.088)	9
Zinc-alpha-2-glycoprotein	9	3.816 (±1.236)	15	3.735 (±0.671)	10
	11				
	12				
	13				
Actin beta	10	1.567 (±0.584)	15	1.913 (±0.883)	10
	16				
	17				
Leukocyte elastase inhibitor	14	0.386 (±0.162)	14	0.201 (±0.126)	9
Carbonic anhydrase VI	15	1.665 (±0.595)	14	2.273 (±0.549)	10
Complement C3	18	0.582 (±0.278)	15	0.626 (±0.268)	10
SPLUNC	19	2.193 (±0.962)	12	1.117 (±0.776)	9
	20				
	21				
Annexin A3	22	0.199 (±0.098)	15	0.133 (±0.091)	8
Ig Kappa chain c region	23	23.031 (±3.968)	15	17.870 (±8.369)	10
Immunoglobulin J chain	24	2.402 (±0.338)	15	1.378 (±1.706)	10
Glutathione S Transferase	25	0.369 (±0.252)	15	0.270 (±0.112)	10
Prolactin inducible protein	26	6.362 (±2.176)	15	4.648 (±1.388)	10
	27				
	28				
	32				
	33				
	34				
Lipocalin -1	29	1.948 (±0.926)	15	1.602 (±0.455)	10
	30				
	31				
	35				
	37				
Interleukin-1 receptor antagonist protein	36	0.041 (±0.016)	15	0.171 (±0.060)	9
Haptoglobin	38	0.397 (±0.246)	15	0.578 (±0.323)	10
	39				
	40				
Transthyretin	41	0.213 (±0.125)	13	0.215 (±0.145)	5
	42				

a volume of a protein expressed as a percentage of the total protein spots in a single particular 2-DE profile;

b RP—rate of presence for particular protein spots in the total 2-DE profiles that were analyzed.

**Table 2:** List of proteins that were differentially expressed.

Proteins	Fold Change a	*p*-Value
PIGR	−1.344	0.008
Plastin 2	+2.381	0
Actin-related protein 3	−5.802	0.001
Leukocyte elastase inhibitor	+1.919	0.004
Carbonic anhydrase VI	−1.365	0.012
Immunoglobulin J chain	+1.743	0
Interleukin-1 receptor antagonist	−4.132	0

a Fold change is the ratio of % volume of T2DM patients with periodontitis to T2DM patients with healthy periodontium.

## References

[1] Wild S.H., Roglic G., Green A., Sicree R., King H. (2004). Global prevalence of diabetes: Estimates for the year 2000 and projections for 2030. Diabetes Care.

[2] Loo J., Yan W., Ramachandran P., Wong D. (2010). Comparative human salivary and plasma proteomes. J. Dent. Res.

[3] The International Diabetes Federation (2011). Diabetes Atlas.

[4] Ministry of Health (2006). 3rd National Health and Morbidity Survey.

[5] Letchuman G., Wan Nazaimoon W., Wan Mohamad W., Chandran L., Tee G., Jamaiyah H., Isa M., Zanariah H., Fatanah I., Ahmad Faudzi Y. (2010). Prevalence of diabetes in the malaysian national health morbidity survey III 2006. Med. J. Malays.

[6] Löe H. (1993). Periodontal disease. The sixth complication of diabetes mellitus. Diabetes Care.

[7] Grossi S.G. (2001). Treatment of periodontal disease and control of diabetes: An assessment of the evidence and need for future research. Ann. Periodontol.

[8] Oliver R.C., Tervonen T. (1994). Diabetes—A risk factor for periodontitis in adults?. J. Periodontol.

[9] Tsai C., Hayes C., Taylor G.W. (2002). Glycemic control of type 2 diabetes and severe periodontal disease in the US adult population. Community Dent. Oral Epidemiol.

[10] Kingman A., Albandar J.M. (2002). Methodological aspects of epidemiological studies of periodontal diseases. Periodontology 2000.

[11] Kinane D., Bouchard P., Group E of European Workshop on Periodontology (2008). Periodontal diseases and health: Consensus report of the sixth European workshop on periodontology. J. Clin. Periodontol.

[12] Grossi S.G., Genco R.J. (1998). Periodontal disease and diabetes mellitus: A two-way relationship. Ann. Periodontol.

[13] Oral Health Division, Ministry of Health, Malaysia (2000). National Oral Health Survey of Adults.

[14] Kaufman E., Lamster I.B. (2002). The diagnostic applications of saliva—A review. Crit. Rev. Oral Biol. Med.

[15] Mandel I.D. (1987). The functions of saliva. J. Dent. Res.

[16] Hofman L.F. (2001). Human saliva as a diagnostic specimen. J. Nutr.

[17] Streckfus C., Bigler L. (2002). Saliva as a diagnostic fluid. Oral Dis.

[18] Wong D.T. (2006). Salivary diagnostics powered by nanotechnologies, proteomics and genomics. J. Am. Dent. Assoc.

[19] Helmerhorst E., Oppenheim F. (2007). Saliva: A dynamic proteome. J. Dent. Res.

[20] Jessie K., Hashim O.H., Rahim Z.H.A. (2008). Protein precipitation method for salivary proteins and rehydration buffer for two-dimensional electrophoresis. Biotechnology.

[21] Gonçalves L.D.R., Soares M.R., Nogueira F., Garcia C., Camisasca D.R., Domont G., Feitosa A.C.R., Pereira D.A., Zingali R.B., Alves G. (2010). Comparative proteomic analysis of whole saliva from chronic periodontitis patients. J. Proteomics.

[22] Huang C.M. (2004). Comparative proteomic analysis of human whole saliva. Arch. Oral Biol.

[23] Jessie K., Pang W.W., Rahim Z.H.A., Hashim O.H. (2010). Proteomic analysis of whole human saliva detects enhanced expression of interleukin-1 receptor antagonist, thioredoxin and lipocalin-1 in cigarette smokers compared to non-smokers. Int. J. Mol. Sci.

[24] Rao P.V., Reddy A.P., Lu X., Dasari S., Krishnaprasad A., Biggs E., Roberts C.T., Nagalla S.R. (2009). Proteomic identification of salivary biomarkers of type-2 diabetes. J. Proteome Res.

[25] Muscari A., Antonelli S., Bianchi G., Cavrini G., Dapporto S., Ligabue A., Ludovico C., Magalotti D., Poggiopollini G., Zoli M. (2007). Serum C3 is a stronger inflammatory marker of insulin resistance than C-reactive protein, leukocyte count, and erythrocyte sedimentation rate. Diabetes Care.

[26] Johansen F., Braathen R., Brandtzaeg P. (2000). Role of J chain in secretory immunoglobulin formation. Scand. J. Immunol.

[27] Max E.E., Korsmeyer S.J. (1985). Human J chain gene. Structure and expression in B lymphoid cells. J. Exp. Med.

[28] Zikan J., Novotny J., Trapane T.L., Koshland M.E., Urry D.W., Bennett J.C., Mestecky J. (1985). Secondary structure of the immunoglobulin J chain. Proc. Natl. Acad. Sci. USA.

[29] Asano M., Komiyama K. (2011). Polymeric immunoglobulin receptor. J. Oral Sci.

[30] Bostanci N., Heywood W., Mills K., Parkar M., Nibali L., Donos N. (2010). Application of label-free absolute quantitative proteomics in human gingival crevicular fluid by LC/MSE (gingival exudatome). J. Proteome Res.

[31] UniProtKB Plastin-2 P13796 (PLSL_HUMAN) http://www.uniprot.org/uniprot/P13796.

[32] Jones S.L., Wang J., Turck C.W., Brown E.J. (1998). A role for the actin-bundling protein L-plastin in the regulation of leukocyte integrin function. Proc. Natl. Acad. Sci. USA.

[33] Grant M.M., Creese A.J., Barr G., Ling M.R., Scott A.E., Matthews J.B., Griffiths H.R., Cooper H.J., Chapple I.L.C. (2010). Proteomic analysis of a noninvasive human model of acute inflammation and its resolution: The twenty-one day gingivitis model. J. Proteome Res.

[34] UniProtKB Leukocyte elastase inhibitor P30740 (ILEU_HUMAN).

[35] Remold-O’Donnell E., Chin J., Alberts M. (1992). Sequence and molecular characterization of human monocyte/neutrophil elastase inhibitor. Proc. Natl. Acad. Sci. USA.

[36] Kivelä J., Parkkila S., Parkkila A.K., Leinonen J., Rajaniemi H. (1999). Salivary carbonic anhydrase isoenzyme VI. J. Physiol.

[37] Jiang W., Gupta D. (1999). Structure of the carbonic anhydrase VI (CA6) gene: Evidence for two distinct groups within the alpha-CA gene family. Biochem. J.

[38] Karhumaa P., Leinonen J., Parkkila S., Kaunisto K., Tapanainen J., Rajaniemi H. (2001). The identification of secreted carbonic anhydrase VI as a constitutive glycoprotein of human and rat milk. Proc. Natl. Acad. Sci. USA.

[39] Kivelä J., Laine M., Parkkila S., Rajaniemi H. (2003). Salivary carbonic anhydrase VI and its relation to salivary flow rate and buffer capacity in pregnant and non-pregnant women. Arch. Oral Biol.

[40] UniProtKB Actin-related protein 3 P61158 (ARP3_HUMAN).

[41] Dayel M.J., Holleran E.A., Mullins R.D. (2001). Arp2/3 complex requires hydrolyzable ATP for nucleation of new actin filaments. Proc. Natl. Acad. Sci. USA.

[42] Dayel M.J., Mullins R.D. (2004). Activation of Arp2/3 complex: Addition of the first subunit of the new filament by a WASP protein triggers rapid ATP hydrolysis on Arp2. PLoS Biol.

[43] Aksentijevich I., Masters S.L., Ferguson P.J., Dancey P., Frenkel J., van Royen-Kerkhoff A., Laxer R., Tedgård U., Cowen E.W., Pham T.H. (2009). An autoinflammatory disease with deficiency of the interleukin-1–receptor antagonist. N. Engl. J. Med.

[44] Arend W.P., Guthridge C.J. (2000). Biological role of interleukin 1 receptor antagonist isoforms. Ann. Rheum. Dis.

[45] Gabay C., Smith M., Eidlen D., Arend W.P. (1997). Interleukin 1 receptor antagonist (IL-1Ra) is an acute-phase protein. J. Clin. Investig.

[46] Granowitz E.V., Clark B., Mancilla J., Dinarello C. (1991). Interleukin-1 receptor antagonist competitively inhibits the binding of interleukin-1 to the type II interleukin-1 receptor. J. Biol. Chem.

[47] Juge-Aubry C.E., Somm E., Giusti V., Pernin A., Chicheportiche R., Verdumo C., Rohner-Jeanrenaud F., Burger D., Dayer J.M., Meier C.A. (2003). Adipose tissue is a major source of interleukin-1 receptor antagonist. Diabetes.

[48] Larsen C.M., Faulenbach M., Vaag A., Ehses J.A., Donath M.Y., Mandrup-Poulsen T. (2009). Sustained effects of interleukin-1 receptor antagonist treatment in type 2 diabetes. Diabetes Care.

[49] Larsen C.M., Faulenbach M., Vaag A., Vølund A., Ehses J.A., Seifert B., Mandrup-Poulsen T., Donath M.Y. (2007). Interleukin-1–receptor antagonist in type 2 diabetes mellitus. N. Engl. J. Med.

[50] Heukeshoven J., Dernick R. (1985). Simplified method for silver staining of proteins in polyacrylamide gels and the mechanism of silver staining. Electrophoresis.

[51] Benjamini Y., Hochberg Y. (1995). Controlling the false discovery rate: A practical and powerful approach to multiple testing. J. R. Stat. Soc. Ser. B.

